# *In vitro* Methods to Cultivate Spiral Ganglion Cells, and Purification of Cellular Subtypes for Induced Neuronal Reprogramming

**DOI:** 10.3389/fnins.2018.00822

**Published:** 2018-11-13

**Authors:** Steven J. Meas, Koji Nishimura, Mirko Scheibinger, Alain Dabdoub

**Affiliations:** ^1^Department of Laboratory Medicine and Pathobiology, University of Toronto, Toronto, ON, Canada; ^2^Biological Sciences, Sunnybrook Research Institute, Toronto, ON, Canada; ^3^Department of Otolaryngology - Head and Neck Surgery, Kyoto University, Kyoto, Japan; ^4^Department of Otolaryngology/HNS, Stanford University, Stanford, CA, United States; ^5^Department of Otolaryngology - Head and Neck Surgery, University of Toronto, Toronto, ON, Canada

**Keywords:** hearing, regeneration, FACS, protocol, inner ear, glia, neurons, auditory

## Abstract

Hearing loss can develop as a consequence of primary auditory neuron degeneration. These neurons are present within the spiral ganglion of the inner ear and co-exist with glial cells that assist in neuronal maintenance and function. There are limited interventions for individuals with hearing impairment, hence novel biological solutions must be explored. Regenerative strategies can benefit from *in vitro* methods to examine the long-term culture of purified cell populations. The culturing of neuronal, glial, and non-neuronal, non-glial cell types in both neonatal and adult mice is presented along with the whole-organ explant culture of the spiral ganglion. High yields of spiral ganglion glial and non-glial cells were cultured from both neonatal and adult mice. Dissociated spiral ganglion cells from Sox2-EGFP mice were sorted based on EGFP expression using fluorescence activated cell sorting. The EGFP+ fraction included purified glial populations, whereas the EGFP- fraction contained non-glial cells. Purified glial cells could be reprogrammed into induced neurons displaying neuronal markers and morphology at a higher efficiency than non-glial cells. Previous studies have only allowed for the short-term culturing of spiral ganglion cell populations and have placed emphasis on neonatal cells. There has also been a lack of methods able to cultivate pure cell populations. Here, the coupling of transgenic mouse lines, fluorescence activated cell sorting and advanced culture conditions allow cultivation and characterization of neuronal, glial and non-neuronal, non-glial cells from the spiral ganglion. These techniques are used to demonstrate that different spiral ganglion cell subtypes (glial vs. non-glial) display different competencies for direct neuronal reprogramming.

## Introduction

The auditory system is a remarkable feat of evolution that transforms invisible mechanical disturbances in the surrounding air into patterned electrical signals that are perceived as changes in pitch and tone. These waves travel toward the tympanic membrane and are transmitted by a chain of three tiny bones, or ossicles, in the middle ear. This signal is delivered to the oval window of the cochlea, initiating movement of the fluid within the cochlear ducts. The basilar membrane moves according to the unique properties of these mechanical vibrations. On top of the basilar membrane sits the organ of Corti, which is the primary sensory organ for hearing that contains the essential hair cells. When the basilar membrane moves, the tip-links on the apex of the hair cells' cilia experience a shearing motion against the tectorial membrane, opening ion channels that will depolarize the hair cell. This depolarization begins a chain reaction that send chemical signals to the dendrites of the adjacent auditory neurons. The dendrites of the auditory neurons receive this information, pass it through Rosenthal's canal into the spiral ganglion, where the cell bodies are located, to the axons that will ultimately send a rapid signal through the auditory nerve up to the auditory centers of the brainstem. These auditory neurons, henceforth referred to as primary auditory neurons (PAN), are divided into two subtypes; Type I PANs which innervate a single inner hair cell and Type II PANs which innervate multiple outer hair cells. Type I PANs are large, bipolar cells that are myelinated by Schwann cells. Type II PANs are smaller, pseudomonopolar cells that are unmyelinated, and we have recently showed that Type II neurons can be divided into two subtypes (Nishimura et al., 2017). Satellite glia and non-myelinating Schwann cells surround the cell bodies of PANs within the spiral ganglion. They play a similar role to glia within the central nervous system by supporting the functions of neuronal activity (Meas et al., 2018). For a complete review of PANs and their functions, please refer to Dabdoub et al. (2016).

Hearing loss can develop after damage to PANs and this degeneration of PANs is a prominent feature of presbycusis, or age-related hearing loss (Liberman, 2017). PANs do not have the capacity to regenerate, hence once they are lost their hearing function can never be restored. Therefore, it is important to develop a greater understanding of the biological parameters present within the spiral ganglion niche in a controlled environment to support research into regenerative strategies (Meas et al., 2018). Early studies on the inner ear relied on organotypic cultures of the chick embryonic otocyst, which offered researchers insights into the differentiation, growth, and development of inner ear cells in an avian model (Strangeways and Fell, 1926). Subsequent studies were developed to apply these techniques to the more difficult mammalian inner ear, where rat embryonic otocysts could be observed to differentiate into the sensorineural components of the inner ear, including hair cells and neurons (Van De Water and Heywood, 1976). Conditions have since improved to facilitate the experimental identification of the neurotrophic elements that support PAN outgrowth and survival, and innervation of endogenous targets (Van De Water and Ruben, 1983; Anniko and de Water, 2009). Organotypic experiments now include a wide range of studies from transgenic experiments that observe live axon growth (Appler et al., 2013) to synapse regeneration (Wang and Green, 2011). Experiments performed, instead, on dissociated cells developed later and initially involved studying the neurotrophic requirements for cell survival (Lefebvre et al., 1991). Since then, techniques have evolved to study the electrophysiology of inner ear cells (Mo and Davis, 1997) and perform genetic manipulations (Roehm et al., 2008). In either type of spiral ganglion culture, organotypic explant or dissociated cells, both forms have placed emphasis on embryonic, neonatal, or early postnatal animals instead of adult animals. Much has come to be understood regarding the development of PANs, axon growth and neurotrophic requirements, however there is still a dearth of knowledge involving PAN subtypes and how they are established during development, including spontaneous discharge rates, intensity coding, and average activity thresholds. This type of information will be critical for rebuilding circuits from the peripheral auditory system to the brain. In this paper, we describe cutting edge hearing biology techniques to culture and study PANs in whole organ immunohistochemistry, organotypic explant cultures, and dissociated cell cultures. We build upon previous techniques by using transgenic mice to isolate spiral ganglion cell types such as neurons and glia using fluorescence activated cell sorting. We additionally demonstrate the cell culture of the adult spiral ganglion, by adapting published techniques.

## Material and equipment

### Reagents

Amphotericin B (250 μg/mL, Thermo Fisher, 15290018)
Ampicillin (100 mg/mL in Ultra-Pure H_2_O, Fisher Scientific, BP1760-25)
B27 supplement (50x, Life technologies, 17504044)
BDNF (100 μg/mL in Ultra-Pure H_2_O, Peprotech, 450-02)
BSA Fraction V (7.5%, Thermo Fisher Scientific, 15260037)
Ciprofloxacin (10 mg/mL in Ultra-Pure H_2_O, Sigma-Aldrich, 17850)
Collagenase Type I (10 mg/mL in MEM, Worthington Biochemical, LS004196)
D-Glucose (450 mg/mL in Ultra-Pure H_2_O, Gibco, 15023-021)
DMEM/F-12, GlutamaxTM supplement (Life technologies 10565042) supplemented with 5 mL of 1M HEPES (final concentration, 10 mM)
DNase I (10,000 U/mL in MEM, Sigma Aldrich, D5025-15KU)
DPBS (Life technologies, 14190250)
EDTA (UltraPure 0.5 M pH 8.0, Invitrogen, 15575-038)
FBS (Life technologies, 16000036)
Fluoromount-G (Southern Biotech, 0100-01)
hbFGF (250 μg/mL in Ultra-Pure H_2_O, Sigma-Aldrich, F0291)
HBSS (Life technologies, 14025092) supplemented with 5 mL of 1 M HEPES (final concentration, 10 mM)
HBSS without calcium and magnesium, without phenol red (Wisent, 311-512-CL) supplemented with 5 mL of 1 M HEPES (final concentration, 10 mM)
HEPES (1 M Gibco, 15630-080)
hEGF (100 μg/mL in Ultra-Pure H_2_O, Sigma-Aldrich, E9644)
Lipofectamine LTX (Life technologies, 15338100)
MEM (Life technologies, 11575-032)
MgCl_2_ (1 M Thermo Fisher, AM9530G)
NT-3 (100 μg/mL in Ultra-Pure H_2_O, Peprotech, 450-03)
N2 supplement (100x, Life technologies, 17502048)
OS-30/Decamethyltetrasiloxane (Corning, 4021768)
Sucrose (Fisher Scientific, BP220-1)
Trypsin-EDTA (0.05%, Life technologies, 25300062)
Trypsin inhibitor from Glycine max (Soybean, Sigma-Aldrich, T6522).

### Antibodies

Rabbit anti-β-tubulin III (TuJ1, Sigma-Aldrich, T2200-200UL, 1:1,000)
Goat anti-Prox1 (R&D, AF2727, 1:500)
Goat anti-Sox2 Y-17 (Santa Cruz Biotechnology, sc-17320, 1:250)
Goat anti-Sox10 N-20 (Santa Cruz Biotechnology, sc-17342, 1:250)
Rabbit anti-Vglut1 (Synaptic Systems, 135302, 1:5,000).

### Recipes for solutions used in protocol

0.5% Triton-PBS: PBS supplemented with 0.5% Triton-X

- For 500 mL: 497.5 mL of PBS supplemented with 2.5 mL of Triton-X (final concentration, 0.5%)

Digestion Solution: MEM supplemented with 100 μg/mL ampicillin, 1 mg/mL D-glucose, 10 mM MgCl_2_

- For 10 mL: 9.87 mL of MEM supplemented with 10 μL of 100 mg/mL ampicillin (final concentration, 100 μg/mL), 22 μL of 450 mg/mL D-glucose (final concentration, 1 mg/mL), 100 μL of 1M MgCl_2_ (final concentration, 10 mM)

Dissecting Solution: MEM supplemented with 4% FBS, 100 μg/mL ampicillin, 1 mg/mL D-glucose, 10 mM MgCl_2_

- For 10 mL: 9.47 mL of MEM supplemented with 400 μL of FBS (final concentration, 4%), 10 μL of 100 mg/mL ampicillin (final concentration, 100 μg/mL), 22 μL of 450 mg/mL D-glucose (final concentration, 1 mg/mL), 100 μL of 1M MgCl_2_ (final concentration, 10 mM)

Explant Media: DMEM/F-12 with Glutamax supplemented with 1x B27 supplement, 1x N2 supplement, Amphotericin B 2.5 μg/mL, Ciprofloxacin 10 μg/mL

- For 10 mL: 9.59 mL of DMEM/F-12 with Glutamax supplemented with 200 μL of 50x B27 (final concentration, 1x), 100 μL of 100x N2 (final concentration, 1x), 100 μL of 250 μg/mL Amphotericin B (final concentration, 2.5 μg/mL), 10 μL of 10 mg/mL ciprofloxacin (final concentration, 10 μg/mL)

Blocking Solution: 0.5% Triton-PBS supplemented with 10% donkey serum, 0.1% BSA

- For 900 μL: 898 μL of 0.5% Triton-PBS supplemented with 90 μL of donkey serum (final concentration, 10%), 12 μL of 7.5% BSA (final concentration, 0.1%)

Centrifugation Solution: Composed of equal parts of (A) and (B). For example, to make 50 mL, add 25 mL of solution (A) and 25 mL of solution (B)

(A) Distilled water supplemented with 0.3 g/mL of sucrose and 0.05% HBSS
  -For 50 mL: Fill a 50 mL centrifuge tube with 15 g of sucrose and 2.5 mL of HBSS. Top up to 50 mL with distilled water (about 33 mL)
(B) Dissecting Solution

Sorting Solution: HBSS without CaCl_2_ and MgCl_2_ supplemented, 1 mM EDTA and 0.1% BSA

- For 9 mL: 8.97 mL of HBSS without CaCl_2_ and MgCl_2_ supplemented with 18 μL of 0.5M EDTA (final concentration, 1mM), 12 μL of 7.5% BSA (final concentration, 0.1%)

Astromedia: DMEM/F-12 with Glutamax supplemented with 10% FBS, 1x B27, 2.5 μg/mL Amphotericin B, 10 μg/mL ciprofloxacin, 10 ng/mL hEGF, 100 ng/mL hbFGF

- For 10 mL: 8.69 mL of DMEM/F-12 with Glutamax supplemented with 1 mL of FBS (final concentration, 10%), 200 μL of 50x B27 (final concentration, 1x), 100 μL of 250 μg/mL Amphotericin B (final concentration, 2.5 μg/mL), 10 μL of 10 mg/mL ciprofloxacin (final concentration, 10 μg/mL), 1 μL of 10 μg/mL hEGF (final concentration, 10 ng/mL), 4 μL of 250 μg/mL hbFGF (final concentration, 100 ng/mL)

Differentiation Media: DMEM/F-12 with Glutamax supplemented with 1x B27, 1x N2, 2.5 μg/mL Amphotericin B, 10 μg/mL ciprofloxacin, 10 ng/mL BDNF, 10 ng/mL NT-3

- For 10 mL: 9.6 mL of DMEM/F-12 with Glutamax supplemented with 200 μL of 50x B27 (final concentration, 1x), 100 μL of 100x N2 (final concentration, 1x), 100 μL of 250 μg/mL Amphotericin B (final concentration, 2.5 μg/mL), 10 μL of 10 mg/mL ciprofloxacin (final concentration, 10 μg/mL), 1 μL of 100 μg/mL BDNF (final concentration, 10 ng/mL), 1 μL of 100 μg/mL NT-3 (final concentration, 10 ng/mL).

### Equipment/material

10 mm or 20 mm uncoated glass bottom dishes (MatTek P35G-1.5-20-C or P35G-1.5-10-C)
15 and 50 ml centrifuge tube
1.5 and 2.5 ml microcentrifuge tubes
35 mm × 10 mm culture dish (Fisher Scientific, 08-772A)
40 μm cell strainer (Fisher Scientific, 22-363-547)
60 mm × 15 mm culture dish (Fisher Scientific, 08-772F)
6-Well Poly-D-Lysine Multiwell Corning Biocoat (Fisher Scientific, 08-774-123)
Fine forceps (FST, Dumont #4 11242-40 and #5 11252-20)
Micro curette (FST, 10080-05)
Surgical scissors (FST, 14090-09)
Sylgard bottom dissection dish
Water bath

Mice: Tau-EGFP knock-in mice (Tucker et al., 2001) (Jackson Laboratories, STOCK *Mapt*^*tm*1(*EGFP*)*Klt*^/J; stock number, 004779) and Sox2-EGFP knock-in mice (Arnold et al., 2011) (Jackson Laboratories, STOCK 129S-Sox2^tm2Hoch^/J; stock number, 017592), which replace the *Mapt* or *Sox2* open reading frame with the EGFP coding sequence, and CD-1 mice (Charles River) were used. Mice were considered neonatal between P0-P2 and adult after P30. Care and euthanasia of the mice used in this study were approved by Sunnybrook Research Institute Animal Care Committee, in accordance with IACUC regulations.

DNA constructs: We used a bi-cistronic expression vector pIRES2 DsRed-Express2 (Clontech), which allowed the simultaneous expression of our protein of interest and DsRed-Express2. pCMV-Ascl1-DsRed2 was constructed by inserting the coding DNA sequence of into the multiple cloning site of the pIRES2 DsRed-Express2 plasmid. Henceforth, referred to as Ascl1-DsRed. We used the parent construct as a negative control, referred to as Empty-DsRed.

### Stepwise procedure

#### Dissection of the inner ear

##### Before dissection

1. At least 1 h before, prepare Matrigel coated dishes. Add 4 mL of DMEM/F-12 with Glutamax to 150 μL of frozen Matrigel and mix thoroughly.
  ^*^Note—work with Matrigel quickly to prevent polymerization.
2. Add 400 μL of Matrigel and DMEM/F-12 mix per 20 mm glass-bottomed dishes and place in 37°C incubator, 5% CO_2_ for at least 1 h before seeding cells.
3. Sterilize forceps, scissors and curette with 70% ethanol prior to use.

##### Dissection

**Figure 1:**
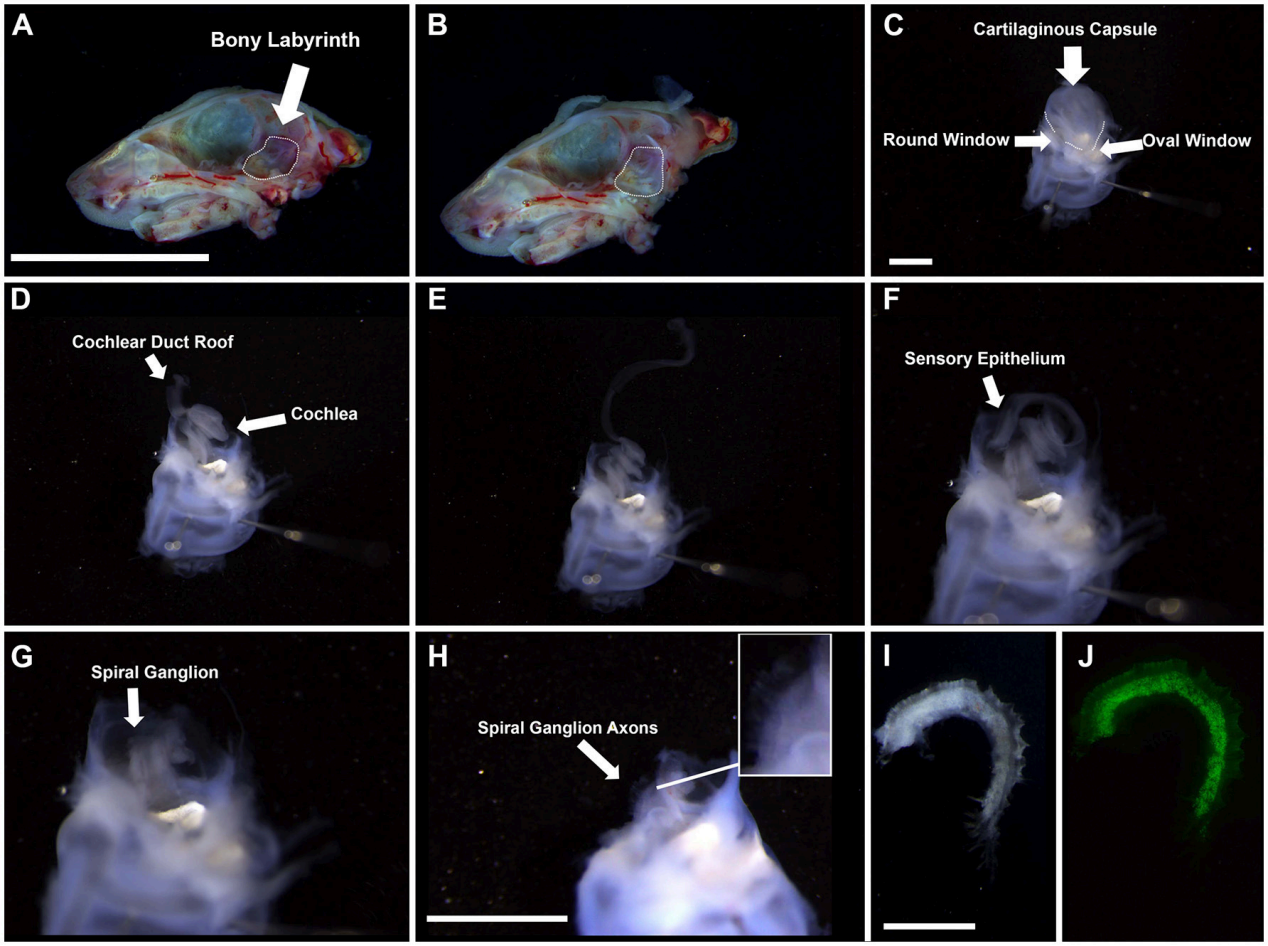
Gross dissection of the spiral ganglion. **(A,B)** Interior view of the neonatal skull after heads were split along the midline and the brain was removed. Dotted line highlights the bony labyrinth within the temporal bone of the right hemisphere. Scale bar; 10 mm **(C)** Appearance of the harvested bony labyrinth after removal of extraneous tissue. Top half contains the cartilaginous capsule surrounding the cochlea. Bottom half contains the semicircular canals of the vestibular system. Scale bar; 1 mm **(D–F)** Removal of cochlear duct roof and sensory epithelium from the modiolus. **(G,H)** Appearance of the spiral ganglion. Axons projecting from the spiral ganglion neurons are visible (see inset). Scale bar; 1 mm **(I,J)** Magnified portion of the spiral ganglion after removal from the modiolus. I, bright field. J, Tau-EGFP. Scale bar; 100 μm.

1. Quickly decapitate pups (P0-P2) with surgical scissors and place on ice. For adult mice euthanize animals in a CO_2_ chamber based on institutional guidelines and perform cervical dislocation prior to decapitation.
2. Using surgical scissors cut along the midline of the head to split it into two halves and scoop out the brain. Place in a culture dish with chilled HBSS.
3. Under a dissecting microscope, isolate the temporal bone in chilled HBSS (Figures 1A,B).
4. Remove the bony labyrinth with both cochlear and vestibular systems intact and place in a Sylgard bottom dissecting dish in dissecting solution.
5. Stabilize the bony labyrinth by inserting two pins through the vestibular system (Figure 1C).
  ^*^Note—for adult tissue, pins will not penetrate the bone and so must be held with forceps.
6. Place the ends of the forceps in both the round and oval windows. Snip.
7. Continue snipping around the cartilaginous membrane until it can be easily removed without damaging the rest of the cochlea. For adult tissue, use coarse forceps to chip away at the bone slowly to maintain the integrity of the tissue.
8. Peel back the cartilaginous membrane and, using forceps, gently sheer the inside to detach the roof of the duct (comprised of the lateral wall, including the stria vascularis and spiral ligament) from the cartilaginous membrane. Remove the cartilage from the cochlea. (Figure 1D)
9. Pinch the roof of the duct at the base of the cochlea and gently tease away from the rest of the cochlea. For adult tissue, the roof is normally removed with the bony capsule. (Figure 1E)
10. Pinching the base of the cochlea, separate the sensory epithelium from the spiral ganglion and carefully tease them apart. Gentle snipping may be necessary to pull the two structures apart. (Figure 1F)
11. Snip the basal portion of the spiral ganglion to remove it from the base of the modiolus (the central pillar which the cochlea wraps) and snip the apical portion to separate the spiral ganglion from the apex of the modiolus. Gently grasping the forceps around the modiolus, tease upwards (basal to apical motions) to separate the spiral ganglion from the modiolus (Figures 1G–J). For adult tissue, due to ossification of surrounding tissue, it is better to remove the spiral ganglion from the modiolus using gentle outward scraping motions.
12. Place the spiral ganglion in a curette.

- For explant culture: remove media from dishes containing Matrigel and replace with 400 μL of DMEM/F-12 with Glutamax. Place spiral ganglia directly onto dish and follow “Culturing of whole spiral ganglion explants” below.
- For dissociation: place spiral ganglia in 400 μL of chilled digestion solution in a 1.5 mL microcentrifuge tube. Follow “Dissociation for primary cell culture” depending on use of neonatal or adult animals below.

##### Troubleshooting points

- Be aware that this is a difficult dissection. However, for cellular dissociation it is not necessary to acquire the whole spiral ganglion in a single piece. Many attempts may be required to attain the skill necessary for whole explants.
- When dissecting the bony labyrinth from the skull it is necessary to ensure that the structure is not broken. This makes the following dissection very difficult.
-Pay particular attention when removing the sensory epithelium from the spiral ganglion. If teasing movements are too forceful then the spiral ganglion risks detaching with the sensory epithelium. This is especially true with adult tissue since bone makes it even more difficult to distinguish the two structures. If there is difficulty locating the spiral ganglion, attempt dissection using a reporter mouse such as Tau-EGFP (as shown in Figure 1J), this facilitates the localization of the spiral ganglion and dissection.

#### Processing of the spiral ganglion

##### Culturing whole spiral ganglion explants

1. Under a stereomicroscope, orient the spiral ganglia so that it forms a semicircular shape as seen in Figures 1I,J or as desired.
2. Slowly aspirate media from the glass-bottomed dishes containing spiral ganglia, leaving only 10 μL remaining. Be careful not to damage or remove spiral ganglia in the process.
3. Once media is removed, allow tissue to settle for 2–3 min (do not exceed 5 min) at room temperature undisturbed.
4. Add explant media drop-wise using a 200 μL micropipettor directly onto tissue very slowly until 400 μL has been added to each well.
5. Carefully transport cultures to a 37°C, 5% CO_2_ incubator and change half of the media every other day. Explants can be reliably preserved for up to 2 weeks.

##### Troubleshooting points

- Whole spiral ganglion tissue is very sensitive to disturbances. If the tissue does not attach, attempt again. Experiment incubating the tissue for different lengths of time after aspirating the media. A lamp may be used to warm tissue and help excess media to evaporate, however do not completely dry tissue. It is also important to use room-temperature media instead of chilled media.

##### Dissociation for primary cell culture

###### Dissociation of neonatal tissue

1. Centrifuge the sample containing whole spiral ganglia at 300 × g for 5 mins at 4°C
2. Discard the supernatant and add 50 μL of pre-warmed 0.5% trypsin-EDTA. Do not triturate. Place in 37°C water bath for 10 min.
3. Add 50 μL of trypsin inhibitor and 100 μL of PBS to the solution and then triturate 20 times with a 200 μL micropipette to completely resuspend the pellet.
4. Pass the resuspended solution through a 40 μm cell strainer onto a 60 × 15 mm culture dish. Using a 200 μL micropipette, transfer the solution to a 1.5 mL microcentrifuge tube. Centrifuge the sample at 300 x g for 5 min at 4°C.
5. Remove the supernatant, transfer the solution to a 15 mL tube and add the necessary amount of astromedia. On average, two spiral ganglia are sufficient to reach confluency within 4 days in a 2% Matrigel coated 20 mm glass bottomed-dish that can hold 400 μL of media (e.g., if eight spiral ganglia were harvested add 3.6 mL of astromedia).
  ^*^Note—if a pellet is not visible remove the top half of the supernatant and top up with astromedia.
6. Add the solution to each dish and very gently shake the dish forward and backwards then left and right to evenly distribute the cells. Let sit for 1 min and then place in the 37°C incubator. The next day, wash plates once with PBS before adding fresh pre-warmed astromedia. Thereafter, change half the astromedia every other day.

##### Troubleshooting points

- Cells should attach and be visible the day after plating, if no cells are observed ensure that cells are not being lost between centrifugation steps. After completely resuspending the pellet transfer 10 μL of solution to another microcentrifuge tube and add 10 μL of trypan blue. Count cells using a hemocytometer.

##### Dissociation of adult tissue (adapted from Lee et al., 2016)

1. In a 1.5 mL microcentrifuge tube, add 50 μL of DNase I, 50 μL of collagenase type I. Incubate at 37°C for 15 min, shaking every 2 min.
2. Add 50 μL of 2.5% trypsin. Incubate at 37°C for 10 min, shaking every 2 min.
3. Add 400 μL of FBS. Using a 1,000 μL micropipette tip, triturate 100 times. After trituration there will still be fragments of bone and tissue visible in the solution.
4. Pass the solution through a 40 μm cell strainer on to the surface of a 60 × 15 mm culture dish. Transfer the filtrate to a 15 mL tube containing 4 mL of centrifugation solution.
5. Centrifuge the 15 mL tube at 200 × g for 5 min at 4°C.
6. Remove supernatant up to the 2 mL mark and add pre-warmed astromedia as necessary.
7. Divide the solution evenly in the wells of a 6-well plate, depending on the number of animals dissected. For 10 animals (twenty spiral ganglia), after plating into three wells, each well of the plate should achieve 90% confluency after 2 weeks. The next day, wash plates once with PBS before adding fresh pre-warmed astromedia. Thereafter, change half the astromedia every other day.
8. To replate cells onto Matrigel-coated 20 mm glass-bottomed dishes, wash wells twice with 2 mL of pre-warmed PBS.
9. Remove the PBS and add 2 mL of pre-warmed 0.05% trypsin/EDTA. Incubate at 37°C for 10 min. At the 5-min mark observe cells under a brightfield microscope and shake the plate gently if cells have not yet detached. Place back in the incubator for the remainder of the 10 min.
10. Add 2 mL of DMEM/F-12 with Glutamax supplemented with 200 μL of FBS (final concentration, 10%) to inactivate the enzymatic reaction and triturate five times with a 1,000 μL micropipette.
11. Add 5 mL of DMEM/F-12 with Glutamax supplemented with 500 μL of FBS (final concentration, 10%) to a 50 mL tube. Pass the sample through a 40 μm cell strainer directly into this 50 mL tube.
12. Centrifuge cells at 200 × g for 10 min at 4°C. Remove supernatant. If there is no pellet only remove up to the 2 mL mark.
13. Resuspend remaining solution in astromedia and add 400–500 μL of solution to each 20 mm glass-bottomed dish. The next day, wash plates once with PBS before adding fresh pre-warmed astromedia. Thereafter, change half the astromedia every other day.

##### Troubleshooting points

- Unlike neonatal cells, adult cells will need to proliferate before becoming obvious. For the first few days, there will be many dead cells and bone debris, which conceal the cells. However, if after a week no cells are visible ensure that cells are not being lost after any point. After completely resuspending the “pellet” transfer 10 μL of solution to another microcentrifuge tube and add 10 μL of trypan blue. Count cells using a hemocytometer. Be very careful when removing supernatant since a pellet is usually not visible using adult tissue.

#### Dissociation for fluorescence activated cell sorting

**Figure 2:**
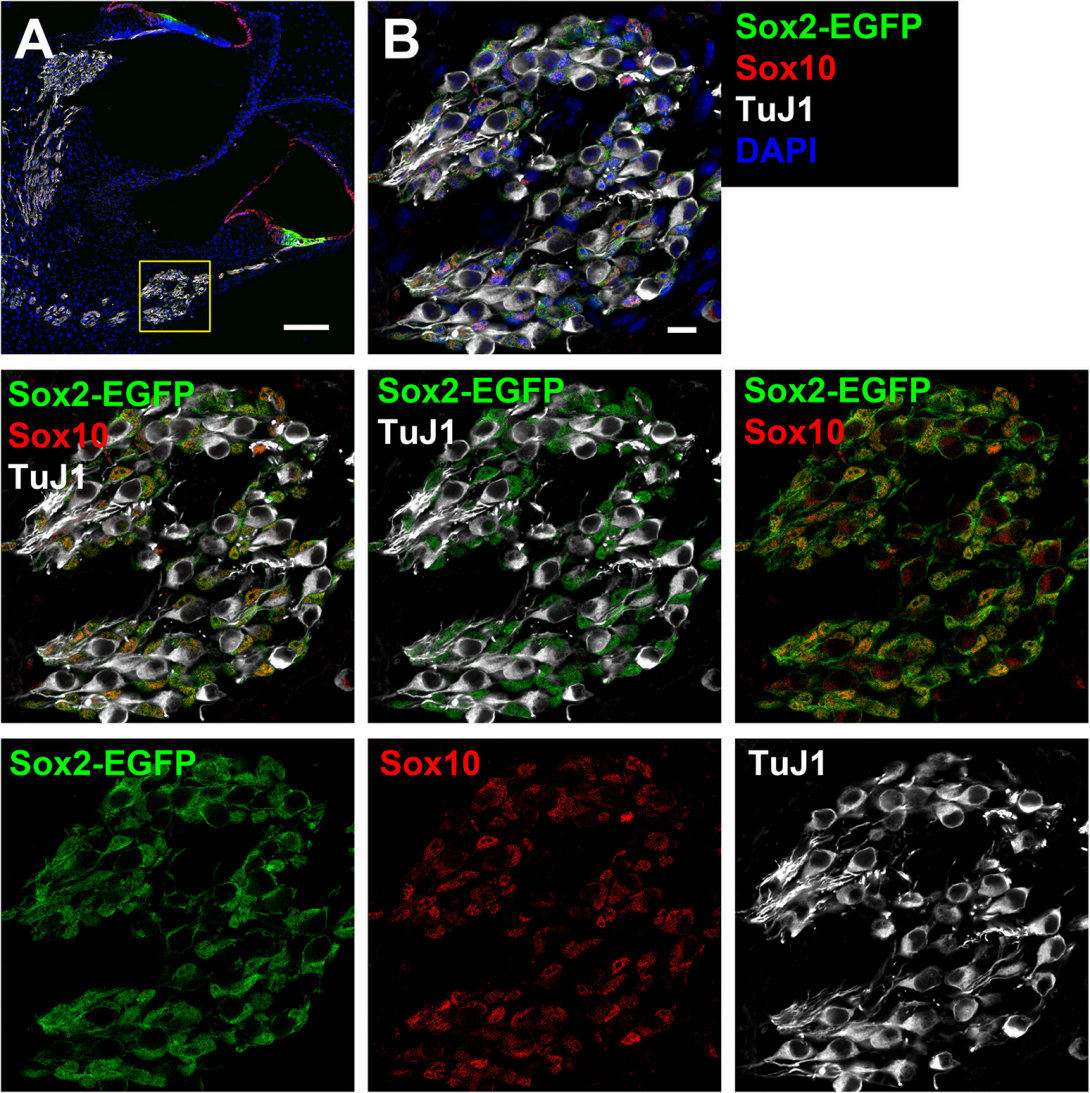
Cross-section of the cochlea harvested from a Sox2-EGFP mouse. **(A)** Two turns of a Sox2-EGFP mice cochlear cross-section can be observed. Sox2-EGFP can be observed within the support cell region of the organ of Corti and the glial cells of the spiral ganglion. The inset refers to images in B. Scale bar; 100 μm **(B)** A zoomed in portion indicating the spiral ganglion and expression of Sox2-EGFP (green; glial marker), Sox10 (red; glial marker), and TuJ1 (white; neuronal marker). Only glia are observed to be EGFP positive in the spiral ganglion as observed by overlap with Sox10, but not TuJ1. Scale bar; 10 μm.

1. Samples for sorting may be acquired directly after dissociation (neonatal cells) or trypsinized after culturing on dishes (neonatal and adults). The following protocol proceeds from the point of cellular suspension.
2. Centrifuge sample at 300 × g for 5 min at 4°C. Remove the supernatant.
3. Resuspend the pellet in 500 μL of sorting solution.
4. Sort cells in a flow cytometer with a 100 μm nozzle, distinct populations can be isolated based on fluorescence. In this paper, we sort based on intensity of EGFP fluorescence based on transgenic mouse lines (Figure 2) or DsRed fluorescence to detect transfection. Cells were sorted with the FACSVantage flow cytometer, operated using the FACSDiva software (BD Biosciences). Sorted cells were collected in Eppendorf low-bind tubes containing 100 μL of sorting solution.
5. Sorted cells can be used for further processing (e.g., RNA extraction) or for culturing. For culturing, pre-fill wells with astromedia (400 μL for 20 mm glass-bottomed dish and 2 mL for 6-well plate) and then evenly add sorted solution directly to wells. Shake gently to plate cells evenly.

##### Troubleshooting point

- To ensure optimal sorting parameters it is necessary to test different sorting conditions on individual user systems. Try higher nozzle diameters and lower pressure to collect larger, more fragile cells. When sorting for EGFP or DsRed fluorescence it is often useful to prepare a control sample containing EGFP-only cells or DsRed-only cells, so the sorting system can become calibrated.

#### Transfection of spiral ganglion non-neuronal cells

##### Transfecting cells using lipofectamine

1. Once cells achieve 70% confluency they are ready to be transfected.
2. Cells were transfected using Lipofectamine LTX based on manufacturer's guidelines. Prepare 50 μL of Opti-MEM supplemented with 1.5 μL of Lipofectamine LTX solution per 20 mm glass-bottomed dish to be transfected. Separately, prepare 50 μL of Opti-MEM supplemented with 500 ng of DNA per 20 mm glass-bottomed dish to be transfected. Incubate for 5 min at room temperature.
3. Combine Lipofectamine and DNA solution together and incubate for 15 min at room temperature.
4. While waiting, wash dishes with 400 μL of PBS. Add 300 μL of pre-warmed Opti-MEM and place in 37°C, 5% CO_2_ incubator.
5. Add 100 μL of Lipofectamine and DNA solution to each dish and incubate for 4 h at 37°C.
6. Wash dishes with 400 μL of PBS and add 400 μL of astromedia. Incubate for 18–24 h in 37°C incubator.
7. The next day, wash cells with 400 μL of PBS and add 400 μL of differentiation media.

#### Immunohistochemistry/immunocytochemistry

^*^Note—for spiral ganglion explants all of the following steps occur at room temperature on a flat surface (no shaking) under a stereomicroscope unless otherwise stated.

1. Remove approximately half of the media and replace it with PBS to wash plates. Repeat twice.
2. Gently remove PBS and replace with 1 mL of 4% formaldehyde-PBS. Incubate for 30 min at room temperature.
3. Remove 4% formaldehyde-PBS and add 1 mL of PBS. Repeat twice. At this point fixed cells can be stored up to 2 weeks, protected from light at 4°C, or processed immediately.
4. Replace solution with 0.5% Triton-PBS. Incubate for 30 min.
5. Replace solution with 0.3 M glycine in 0.5% Triton-PBS. Incubate for 30 min.
6. Replace solution with blocking solution. Incubate for 1 h.
7. Replace with primary antibody solution containing 10% donkey serum in 0.5% Triton-PBS. Antibody concentrations must be optimized to user specifications. Incubate at 4°C overnight.
8. The following day, remove the primary antibody solution and wash cells gently with PBS. Repeat three times. Incubate for 10 min each.
9. Replace solution with secondary antibody solution containing 1% donkey serum in 0.5% Triton-PBS. Incubate for 1 h.
10. Remove secondary antibody solution and wash cells gently with PBS. Repeat three times. Incubate for 10 min each.
11. After washing steps add 750 μL of OS-30/Decamethyltetrasiloxane solvent to the lid of the glass-bottomed dish and place the bottom half into the lid, bottom-side down. Incubate for 1 h.
12. Using a scalpel carefully scrape away excess material from coverslip and mount onto glass slides with Fluoromount-G. Let samples settle overnight.

#### Quantification of induced neuron reprogramming

1. Transfected cells were selected at random based on the expression of the DsRed marker. Images were taken using a Zeiss A.1 Epifluorescence Microscope using the ZEN software. Images were analyzed using ImageJ.
2. These cells were assayed for the expression of neuronal markers βIII-tubulin (TuJ1).
3. Induced neurons were quantified based on the criteria that they expressed a neuronal marker TuJ1 and had at least one extension that was three times longer than its cell body.

### Anticipated results and discussion

**Figure 3:**
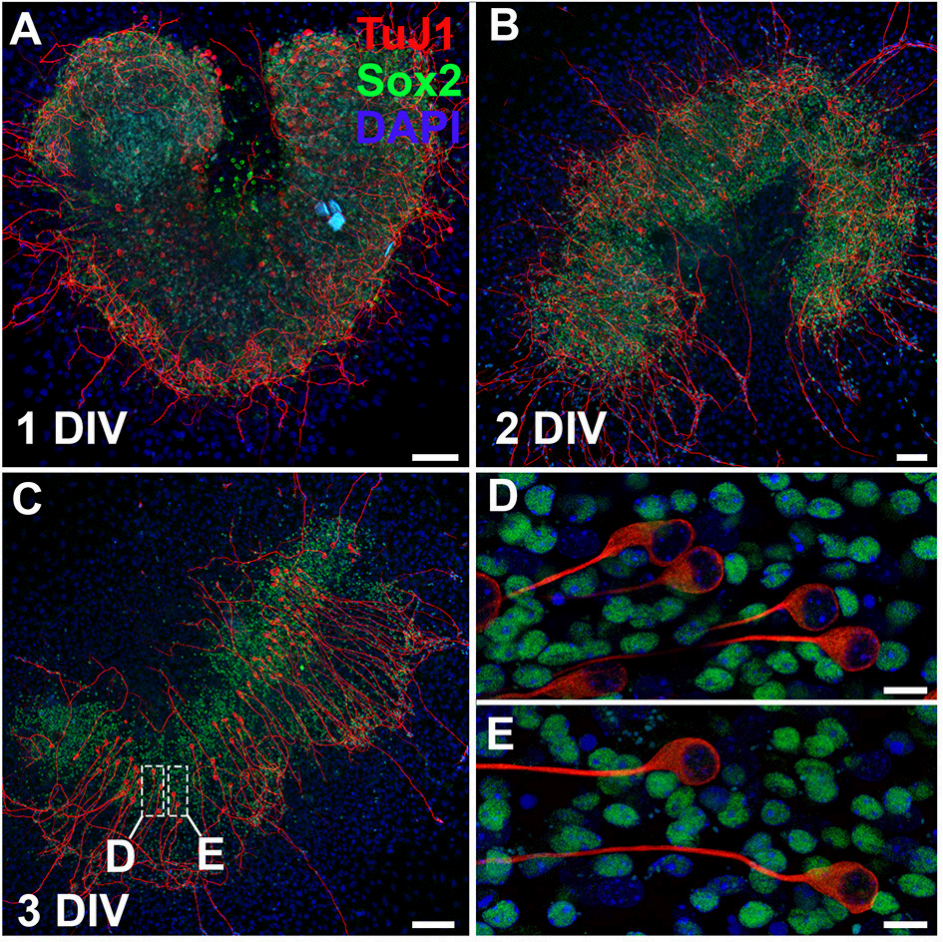
Whole organ explant culture of the spiral ganglion. **(A–C)** Spiral ganglion explants cultured on 2% Matrigel-coated dishes for 1 day **(A)**, 2 days **(B)**, or 3 days **(C)**. Neurons are stained with TuJ1 (red) and glial cells are stained with Sox2 (green) antibodies. Scale bar;100 μm **(D,E)** Magnified images from spiral ganglion explants cultured for 3 days. Neurons are amongst glial cells. Scale bar; 10 μm.

The protocol as outlined presents a detailed overview of methods to explore the intricacies of the spiral ganglion *in vitro*. It is possible to culture whole spiral ganglion explants from postnatal day (P) 0 leading up to P10. Starting at P3, the cartilaginous capsule surrounding the cochlea and the cartilage within the cochlea begin to ossify. At P6, the ossification develops into very brittle tissue that is easily damaged if not careful. After P14, the bone is nearly completely ossified and techniques used to harvest adult cochleae must be used. In this case, we do not recommend culturing explants with excessive bone attached; however, it is possible to culture a portion of the spiral ganglion from the apex of the cochlea from nearly any age. Here we have cultured a spiral ganglion explant extracted from a P0 CD1 mouse and cultured for up to 3 days *in vitro* (DIV) in 10% FBS-DMEM/F12 with Glutamax (Figure 3). After 1 DIV, neurons stained with βIII-tubulin (TuJ1) can be observed extending axons radially outwards. Explants can be cultured for up to 2 weeks. Spiral ganglion explants are especially useful in modeling the organ *ex vivo*, since the spiral ganglion is a restricted niche that makes *in vivo* access difficult. Therefore, studies that intend to study axon pathfinding, growth or regeneration of spiral ganglion neurons are particularly suited for the use of explant cultures (Wang and Green, 2011; Appler et al., 2013). As an example, concentration gradients can be developed using slow-release hydrogels or electrical fields can be generated to observe their effect on spiral ganglion neurons.

**Figure 4:**
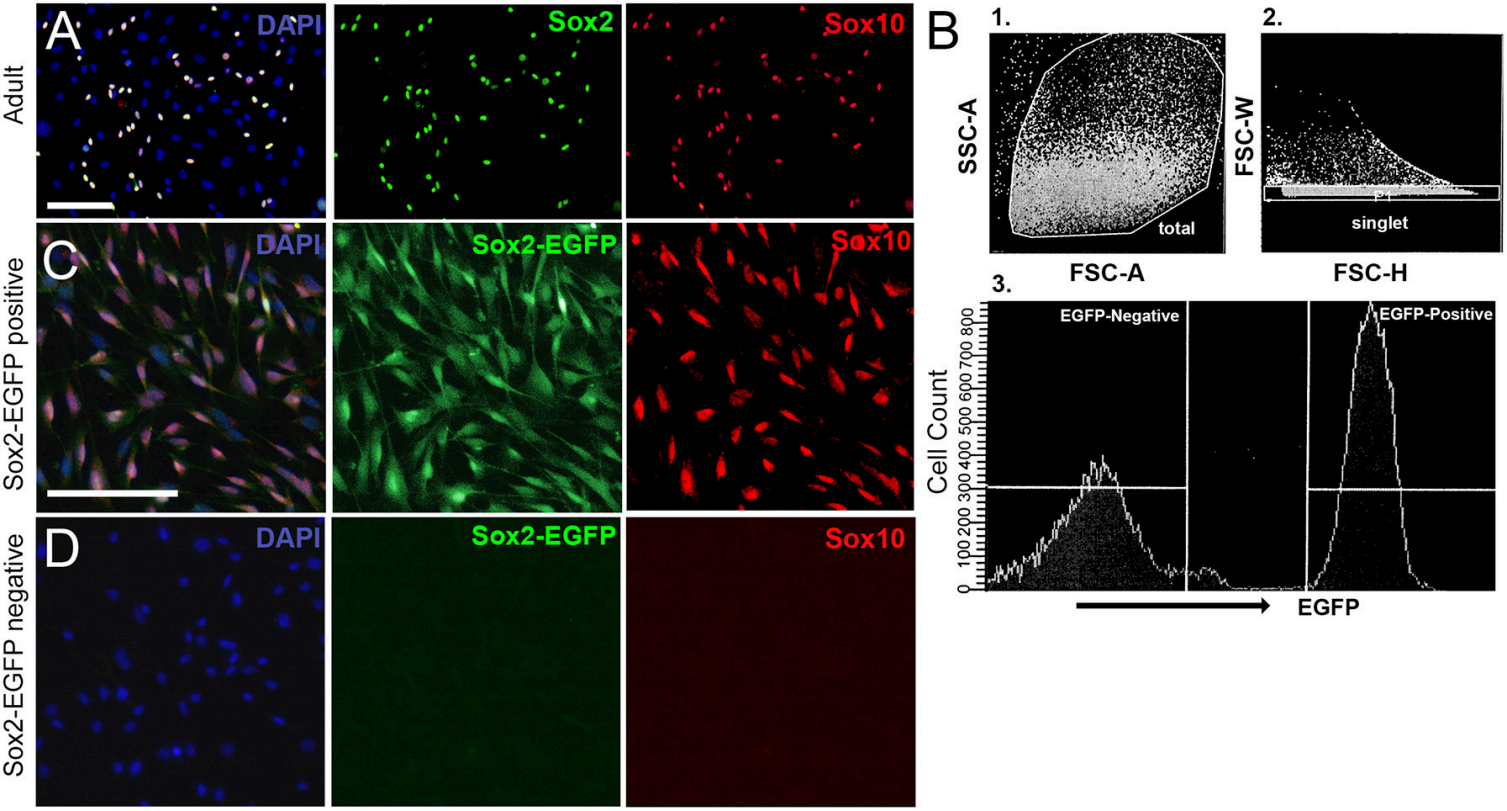
Culturing spiral ganglion glial cells harvested from adult CD1 mice and sorted from neonatal Sox2-EGFP mice. **(A)** Spiral ganglion glial cells harvested from adult CD1 tissue cultured for 3 weeks and re-plated onto 2% Matrigel coated dishes. Glial cells (Sox2, green; Sox10, red) are observed to culture along with other non-neuronal, non-glial cells. Scale bar; 100 μm **(B)** Spiral ganglion single cell suspensions harvested from one-day old Sox2-EGFP mice were analyzed for the expression of EGFP. Representative fluorescence-activated cell-sorting analysis gating strategy is shown. First cells were gated for (1) forward and side scatter of dissociated cells followed by (2) gating for doublet discrimination, and lastly for (3) EGFP expression. A representative histogram with gating of EGFP+ and EGFP- populations is shown. **(C,D)** Spiral ganglion glial cells harvested from P0 Sox2-EGFP tissue and sorted into Sox2-positive and Sox2-negative fractions using a fluorescence activated cell sorter based on EGFP intensity. Glial cells nearly exclusively make up the positive sorted fraction **(B)** as based on Sox2-EGFP and Sox10 expression. No glia were observed in the Sox2-negative fraction. Scale bar; 10 μm.

In some cases, it is more suitable to dissociate tissue into single cells, especially when examining specific populations of cells for survival, regeneration, signaling, transcriptome analyses, or development. Cells cultured from neonatal tissue typically contain a mixture of neuronal, glial, and non-neuronal, non-glial cells (Figure 4). This makes it difficult to perform studies on any single cell type. Fortunately, it is possible to purify populations of desired cells using fluorescence-based sorting of cells extracted from transgenic mouse lines. After excluding debris and dead cells based on size, forward and lateral scattering, the remaining cells were sorted based on the level of EGFP-fluorescence (Figure 4B). In Figures 4C,D, spiral ganglion cells were extracted from P0 Sox2-EGFP mice. At this developmental age, the Sox2-EGFP positive population contained glial cells, whereas the Sox2-EGFP negative population contained non-neuronal, non-glial cells and neuronal cells. There is a slight upregulation of Sox2 in neuronal cells around P0 (Nishimura et al., 2017); however, it may be possible to exclude neurons based on strict gating of EGFP intensity. Alternative cells such as those extracted from Sox10-Venus transgenic mice may be used to harvest neural-crest derived cells such as glia, but not neurons (Wakaoka et al., 2013). Cells extracted from a Tau-EGFP mouse line, on the other hand, can instead be used to isolate a pure population of neuronal cells from the typical mixture of cells found in the spiral ganglion (Noda et al., 2018). Adult cells cannot be sorted based on fluorescence immediately after dissociation, due to low cellular yields, low fluorescence intensity and abundance of bone fragment debris. Cells cultured from adult tissue using our protocol are found to contain a mixture of glial and non-neuronal, non-glial cells, but rarely any neuronal cell type. To culture neurons, please refer to Lee et al. (2016). However, once colonies are expanded on poly-D-lysine coated plates they can be trypsinized and sorted.

**Figure 5:**
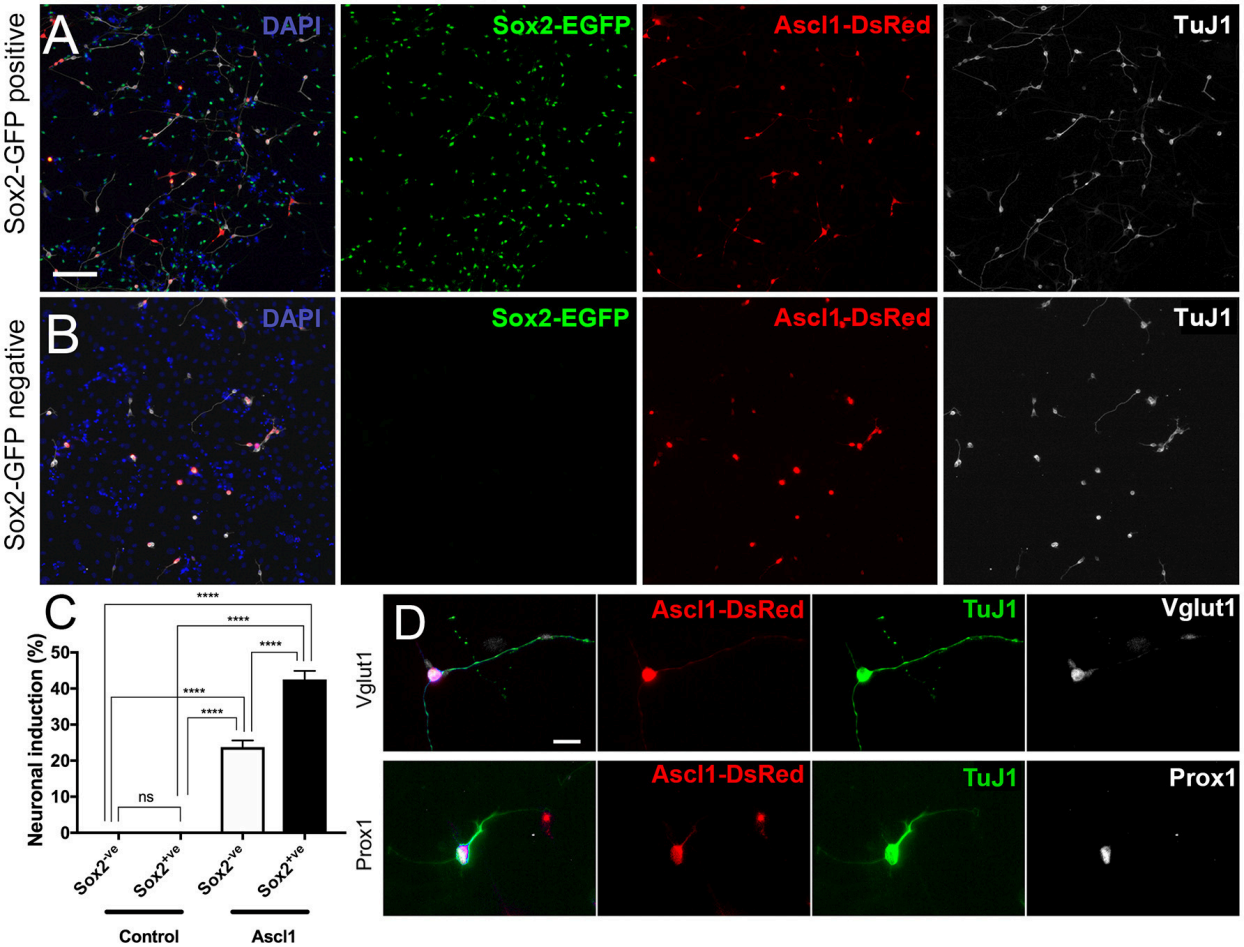
Reprogramming sorted Sox2-EGFP cells into induced neurons. **(A,B)** Cells harvest from P0 Sox2-EGFP mice were sorted based on EGFP intensity and collected into two fractions; Sox2-positive **(A)** and Sox2-negative **(B)**. Cells were transfected with Ascl1-DsRed (red) and fixed after 7 days. Cells in both fractions could become reprogrammed into induced neurons based on TuJ1 (white). Scale bar; 100 μm **(C)** Quantification of neuronal conversion efficiency based on the percentage of DsRed transfected cells that are also TuJ1 positive and have a neurite length at least three times the size of the cell soma. ^****^p < 0.0001. **(D)** Staining of reprogrammed cells displaying expression of additional neuronal markers such as Vglut1 and Prox1. Scale bar; 10 μm.

As an example of potential experimental uses for purified spiral ganglion cell populations, we have included the transfection of EGFP+ and EGFP- populations (referred to as Sox2-positive and Sox2-negative, hereon) with *Ascl1*-*DsRed*, a neurogenic transcription factor, and *Empty-DsRed* (Figures 5A,B). It should be noted that the source of cells for reprogramming is important. Studies performed using astrocytes have discovered that reprogrammed cells developed into induced neurons that were similar to the endogenous neurons from the niche in which they were harvested (Meas et al., 2018). Hence, we are interested in using cells harvested from the spiral ganglion in order to induce neurons that are similar to endogenous PANs. Moreover, those cells used for conversion experiments *in vitro* should be, ideally, the same type of cells used as source of induced neurons *in vivo*. A two-way ANOVA was conducted that examined the effect of genes of transfection and cell types on neuronal conversion efficiency. There was a statistically significant interaction between the effects of genes and cell types, *F*_(1, 20)_ = 27.53, *p* < 0.0001. An analysis of simple main effects indicated that *Ascl1-DsRed* induced neurons at significantly higher efficiency than *Empty-DsRed* [*F*_(1, 20)_ = 343.70, *p* < 0.0001; Ascl1-transfected groups, *n* = 7; Control-transfected groups, *n* = 5]. Additionally, Sox2-positive cells transfected with *Ascl1-DsRed* were more likely to reprogram into induced neurons as compared to Sox2-negative cells [Sox2-positive 44%; Sox2-negative 23%; *F*_(1, 20)_ = 27.53, *p* < 0.0001; Figure 5C]. Multiple comparisons performed using Sidak's method demonstrated *Ascl1*-*DsRed*-induced Sox2-positive cells indicated higher neuronal conversion efficiency than Ascl1-induced Sox2-EGFP negative cells (*p* < 0.0001). The induced cells expressed neuronal markers Vglut1 and Prox1 (Figure 5D). Other transgenic backgrounds can be used to select other subsets of cells. For example, when Tau-EGFP negative cells are transfected with *Ascl1-DsRed*, the neuronal induction process will stimulate the upregulation of *Mapt* (Tau), hence the cells reprogrammed using this background will upregulate EGFP expression. These cells can then be easily identified for electrophysiology or sorted based on Tau-EGFP and DsRed fluorescence. This allows the collection of pure populations of induced neurons, cells that failed to reprogram and untransfected cells (Noda et al., 2018). This can be especially useful for RNAseq to evaluate changes in either type of cell and to offer greater insight into the individual transcriptomic profile of each cell during reprogramming.

In this paper, we have outlined strategies to assess the function and identity of spiral ganglion cells extracted from neonatal and adult mice. This is the first paper to offer detailed methodology to isolate purified populations of spiral ganglion cells into its neuronal, glial, and non-neuronal, non-glial components using transgenic mouse lines, including in adult tissue. The greatest limitation with this technique depends on the researcher's ability to dissect the tissue, hence practice is required for complete mastery. We believe there is enormous potential for these techniques to better understand spiral ganglion growth and function during both development and adulthood.

## Author contributions

SM, KN, and AD: conceptualization; SM, KN, MS, and AD: formal analysis; SM and AD: funding acquisition; SM, KN, MS, and AD: investigation; AD: project administration and supervision; SM, KN, and MS: validation and visualization; SM, KN, MS, and AD: writing—original draft; SM, KN, MS, and AD: writing—review and editing.

### Conflict of interest statement

The authors declare that the research was conducted in the absence of any commercial or financial relationships that could be construed as a potential conflict of interest.

## References

[B1] Anniko M. Van de Water T. R. V. (2009). Synaptogenesis in co-cultured inner ear explants which share a single statoacoustic ganglion. Acta Otolaryngol. 102, 415–422. 10.3109/00016488609119426 3788541

[B2] Appler J. M. Lu C. C. Druckenbrod N. R. Yu W.-M. Koundakjian E. J. Goodrich L. V. (2013). Gata3 is a critical regulator of cochlear wiring. J. Neurosci. 33, 3679–3691. 10.1523/JNEUROSCI.4703-12.2013 23426694 PMC3613247

[B3] Arnold K. Sarkar A. Yram M. A. Polo J. M. Bronson R. Sengupta S.. (2011). Sox2(+) adult stem and progenitor cells are important for tissue regeneration and survival of mice. Cell Stem Cell 9, 317–329. 10.1016/j.stem.2011.09.001 21982232 PMC3538360

[B4] Dabdoub A. Fritzsch B. Popper A. N. Fay R. R. (eds.). (2016). The Primary Auditory Neurons of the Mammalian Cochlea. New York, NY: Springer New York.

[B5] Lee J.-H. Sihn C. Wang W. Flores C. M. P. Yamoah E. N. (2016). *In vitro* functional assessment of adult spiral ganglion neurons (SGNs). Methods Mol. Biol. 1427, 513–523. 10.1007/978-1-4939-3615-1_29 27259946

[B6] Lefebvre P. P. Van De Water T. R. Weber T. Rogister B. Moonen G. (1991). Growth factor interactions in cultures of dissociated adult acoustic ganglia: neuronotrophic effects. Brain Res. 567, 306–312. 10.1016/0006-8993(91)90809-A 1817733

[B7] Liberman M. C. (2017). Noise-induced and age-related hearing loss: new perspectives and potential therapies. F1000Res. 6:927. 10.12688/f1000research.11310.1 28690836 PMC5482333

[B8] Meas S. J. Zhang C.-L. Dabdoub A. (2018). Reprogramming glia into neurons in the peripheral auditory system as a solution for sensorineural hearing loss: lessons from the central nervous system. Front. Mol. Neurosci. 11:77. 10.3389/fnmol.2018.00077 29593497 PMC5861218

[B9] Mo Z. L. Davis R. L. (1997). Endogenous firing patterns of murine spiral ganglion neurons. J. Neurophysiol. 77, 1294–1305. 10.1152/jn.1997.77.3.1294 9084597

[B10] Nishimura K. Noda T. Dabdoub A. (2017). Dynamic expression of Sox2, Gata3, and Prox1 during primary auditory neuron development in the mammalian cochlea. PLoS ONE 12:e0170568. 10.1371/journal.pone.0170568 28118374 PMC5261741

[B11] Noda T. Meas S. J. Nogami J. Amemiya Y. Uchi R. Ohkawa Y.. (2018). Direct reprogramming of spiral ganglion non-neuronal cells into neurons: toward ameliorating sensorineural hearing loss by gene therapy. Front. Cell Dev. Biol. 6:16. 10.3389/fcell.2018.00016 29492404 PMC5817057

[B12] Roehm P. C. Xu N. Woodson E. A. Green S. H. Hansen M. R. (2008). Membrane depolarization inhibits spiral ganglion neurite growth viaactivation of multiple types of voltage sensitive calcium channels and calpain. Mol. Cell Neurosci. 37, 376–387. 10.1016/j.mcn.2007.10.014 18055215 PMC2265381

[B13] Strangeways T. S. P. Fell H. B. (1926). Experimental studies on the differentiation of embryonic tissues growing *in vivo* and *in vitro*.–II. The development of the isolated early embryonic eye of the fowl when cultivated *in vitro*. Proc. R. Soc. B Biol. Sci. 100, 273–283. 10.1098/rspb.1926.0049

[B14] Tucker K. L. Meyer M. Barde Y. A. (2001). Neurotrophins are required for nerve growth during development. Nat. Neurosci. 4, 29–37. 10.1038/82868 11135642

[B15] Van De Water T. R. Heywood P. (1976). The *in vitro* development of innervated sensory hair cells of a mammal. Acta Otolaryngol. 82, 337–342. 10.3109/00016487609120917 998202

[B16] Van De Water T. R. Ruben R. J. (1983). A possible embryonic mechanism for the establishment of innervation of inner ear sensory structures. Acta Otolaryngol. 95, 470–479. 10.3109/00016488309139431 6880656

[B17] Wakaoka T. Motohashi T. Hayashi H. Kuze B. Aoki M. Mizuta K.. (2013). Tracing Sox10-expressing cells elucidates the dynamic development of the mouse inner ear. Hearing Res. 302, 17–25. 10.1016/j.heares.2013.05.003 23684581

[B18] Wang Q. Green S. H. (2011). Functional role of neurotrophin-3 in synapse regeneration by spiral ganglion neurons on inner hair cells after excitotoxic trauma *in vitro*. J. Neurosci. 31, 7938–7949. 10.1523/JNEUROSCI.1434-10.2011 21613508 PMC3132175

